# Favorable outcome after fetal swallowing of a Somatex® intrauterine shunt

**DOI:** 10.1007/s00404-024-07802-w

**Published:** 2024-11-02

**Authors:** A. Rejaey, I. Gottschalk, E. C. Weber, A. Messling, J. Hubertus, C. Berg

**Affiliations:** 1 MVZ Am Marienplatz 2, Witten, Germany; 2 Division of Prenatal Medicine and Fetal Surgery, Department of Obstetrics and Gynecology, University Hospital of Cologne, Köln, Kerpener Str. 34, 50931 Germany; 3 Department of Pediatric Surgery, Ruhr-Universität Bochum, Bochum, Germany

**Keywords:** Fetal surgery, Vesico-amniotic shunt, Neonatal surgery, Congenital urinary tract abnormalities, Shunt complications

## Abstract

This report describes the ingestion of a dislodged Somatex Intrauterine Stent by the fetus. At 35 weeks one shunt was visualized in the fetal stomach, suggesting that the fetus had swallowed it. The shunt kept its position in the stomach until the last follow up scan at 37 weeks. At 38 weeks the patient went into spontaneous labor and vaginally delivered a boy weighting 3590 g. The first chest X-ray on the day of birth demonstrated the dislodged shunt in the duodenum while the other shunt drained the left kidney. The mother started breastfeeding. The x-ray on the second day of life was made after removal of the second shunt and the creation of a nephrostoma and demonstrated the dislodged shunt in the ileum. On the third day of life the shunt was found in the neonate’s stool. The neonate was dismissed on day 7 with antibiotic prophylaxis. To our best knowledge this is the first report of an intrauterine ingestion of a Somatex Intrauterine Stent. It demonstrates that in the intrauterine period the shunt remains in the stomach and starts to pass the bowel after birth, probably prompted by breastfeeding. In our case the shunt was eventually excreted without any damage to the digestive system.

A 32-year-old patient in her 2nd pregnancy was referred to our prenatal unit at 26-week gestation with a large unilateral urinoma. A follow-up scan at 28 weeks demonstrated massive growth of the urinoma measuring 80 × 55 mm which prompted the decision for intrauterine shunting using a Somatex® intrauterine shunt that is 25 mm long with a diameter of 2.3 mm consisting of a nitinol wire mesh and internal impermeable silicone coating. The shunt has self-deploying parasols at both ends and can be placed through an 18-G puncture cannula [1]. The initial shunt insertion failed due to fetal movements and the shunt dislodged in the amniotic cavity. Repeated intervention under fetal anesthesia was successful and resulted in complete resolution of the unilateral urinoma with residual moderate hydronephrosis. Biweekly follow-up scans demonstrated the correct position of the second shunt while the dislodged shunt adhered to the fetal scalp. At 35 weeks, one shunt was visualized in the fetal stomach (Fig. 1) while the other shunt was still draining the left kidney, suggesting that the fetus had swallowed the dislodged shunt. The shunt kept its position in the stomach until the last follow-up scan at 37 weeks. At 38 weeks, the patient went into spontaneous labor and vaginally delivered a boy weighting 3590 g. The first chest X-ray on the day of birth demonstrated the dislodged shunt in the duodenum while the other shunt drained the left kidney. The mother started breastfeeding. The X-ray on the second day of life was made after removal of the second shunt and the creation of a nephrostoma and demonstrated the dislodged shunt in the ileum. On the third day of life, the shunt was found in the neonate’s stool. The neonate was dismissed on day 7 with antibiotic prophylaxis.

**Fig. 1:**
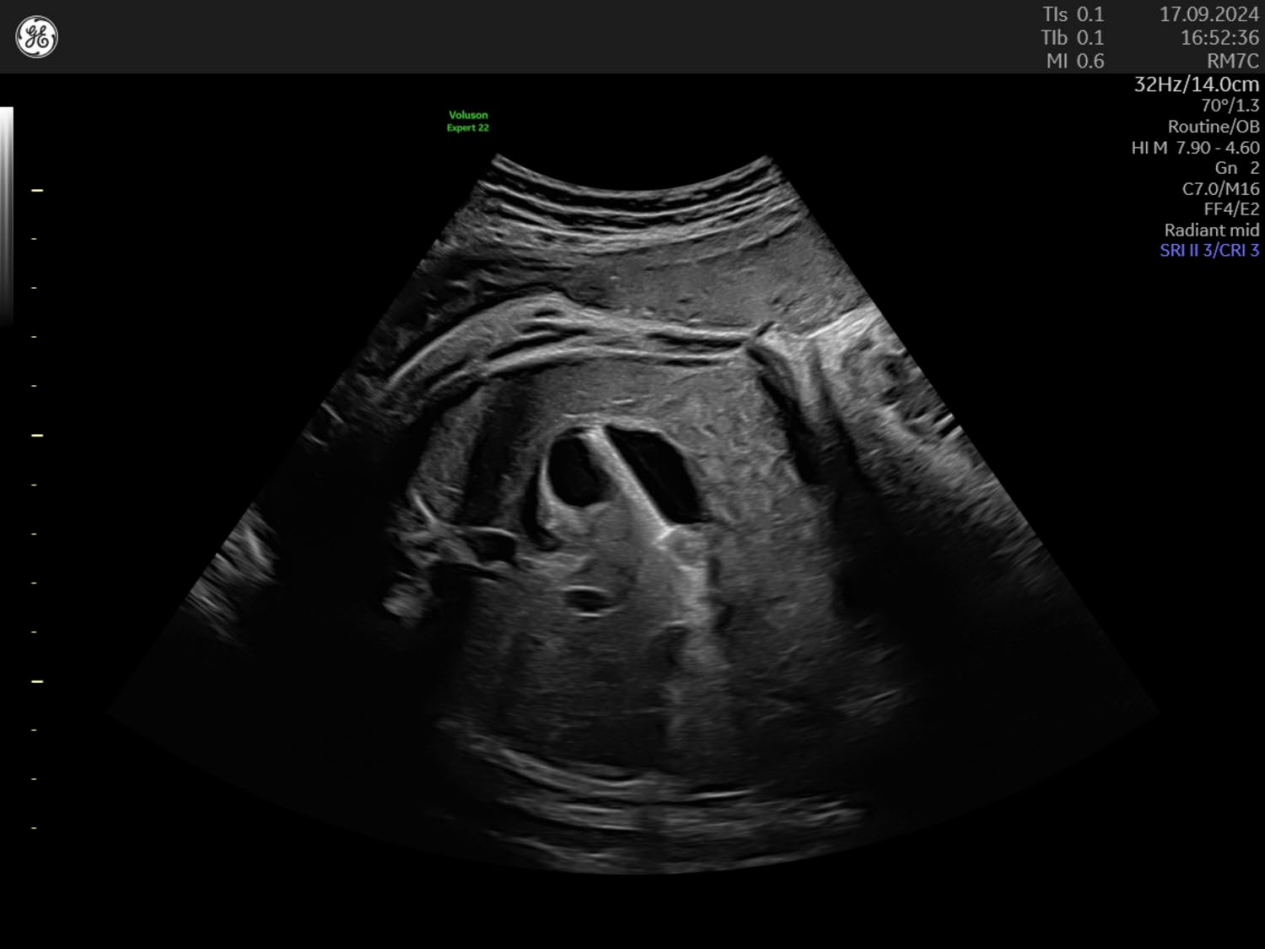
Transverse view of the fetal abdomen at 35-week gestation demonstrating the shunt inside of the fetal stomach

To our best knowledge, this is the first report of an intrauterine ingestion of a Somatex® intrauterine shunt. It demonstrates that in the intrauterine period, the shunt remains in the stomach and starts to pass the bowel after birth, probably prompted by breastfeeding. In our case, the shunt was eventually excreted without any damage to the digestive system. Despite the favorable outcome in our case, it has to be considered that the parasols of the shunt are sharp and might cause damage to the surrounding structures. Therefore, comparable cases should be delivered and followed up in experienced centers until the excretion of the shunt.

## Data Availability

not applicable.

## References

[1] Strizek B, Gottschalk I, Recker F et al (2020) Vesicoamniotic shunting for fetal megacystis in the first trimester with a Somatex® intrauterine shunt. Arch Gynecol Obstet 302:133–140. 10.1007/s00404-020-05598-z 32449061 10.1007/s00404-020-05598-z PMC7266802

